# Can telerehabilitation services combined with caregiver-mediated exercises improve early supported discharge services poststroke? A study protocol for a multicentre, observer-blinded, randomized controlled trial

**DOI:** 10.1186/s12883-021-02533-w

**Published:** 2022-01-17

**Authors:** Marijn Mulder, Corien Nikamp, Rinske Nijland, Erwin van Wegen, Erik Prinsen, Judith Vloothuis, Jaap Buurke, Gert Kwakkel

**Affiliations:** 1 Department of Rehabilitation Medicine, Amsterdam University Medical Centre, location VU University Medical Centre, Amsterdam Movement Sciences, PO Box 7057, 1007 MB Amsterdam, The Netherlands; 2 Amsterdam Rehabilitation Research Centre | Reade, Amsterdam, The Netherlands; 3 Roessingh Research and Development, Enschede, The Netherlands; 4 Department of Biomedical Signals and Systems, Technical Medical Centre, University of Twente, Enschede, the Netherlands; 5 Amsterdam Neuroscience, Vrije Universiteit, Amsterdam, The Netherlands; 6 Department op Biomechanical Engineering, Technical Medical Centre, University of Twente, Enschede, the Netherlands; 7 Department of Physical Therapy and Human Movement Sciences, Northwestern University, Chicago, IL USA; 8 Roessingh, Centre for Rehabilitation, Enschede, The Netherlands

**Keywords:** Stroke, Telerehabilitation, Caregivers, Exercise, Walking, Clinical trial protocol

## Abstract

**Background:**

Recovery of walking ability is an important goal for patients poststroke, and a basic level of mobility is critical for an early discharge home. Caregiver-mediated exercises could be a resource-efficient strategy to augment exercise therapy and improve mobility in the first months poststroke. A combination of telerehabilitation and face-to-face support, blended care, may empower patient-caregiver dyads and smoothen the transition from professional support to self-management. The Armed4Stroke study aims to investigate the effects of a caregiver-mediated exercise program using a blended care approach in addition to usual care, on recovery of mobility in the first 6 months poststroke.

**Methods:**

A multicentre, observer-blinded randomized clinical trial in which 74 patient-caregiver dyads will be enrolled in the first 3 months poststroke. Dyads are randomly allocated to a caregiver-mediated exercises intervention or to a control group. The primary endpoint is the self-reported mobility domain of the Stroke Impact Scale. Secondary endpoints include care transition preparedness and psychological functioning of dyads, length of inpatient stay, gait-related measures and extended ADL of patients, and caregiver burden. Outcomes are assessed at enrolment, end of treatment and 6 months follow-up.

**Results:**

During 8 weeks, caregivers are trained to become an exercise coach using a blended care approach. Dyads will receive a tailor-made, progressive training program containing task-specific exercises focusing on gait, balance, physical activity and outdoor activities. Dyads are asked to perform the training program a minimum of 5 times a week for 30 min per session, supported by a web-based telerehabilitation system with instruction videos and a messaging environment to communicate with their physiotherapist.

**Conclusions:**

We hypothesize that the Armed4Stroke program will increase self-reported mobility and independence in ADL, facilitating an early discharge poststroke. In addition, we hypothesize that active involvement of caregivers and providing support using blended care, will improve the care transition when professional support tapers off. Therefore, the Armed4Stroke program may complement early supported discharge services.

**Trial registration:**

Netherlands Trial Register, NL7422. Registered 11 December 2018.

**Supplementary Information:**

The online version contains supplementary material available at 10.1186/s12883-021-02533-w.

## Background

In 2017, approximately 104 million people were living with the consequences of stroke [1]. Most patients have long-term disabilities and remain dependent on informal caregivers [2]. Stroke rehabilitation is typically front-loaded, with resources mainly focused on inpatient care [3]. After discharge, professional support tapers off and the majority of home-dwelling stroke patients are physically inactive [4]. Caregivers provide sustained support and could promote self-generated physical activity [5]. However, caregivers often feel unprepared for their new caregiving role [6] and nearly half of community-dwelling stroke patients continue to report unmet needs across a range of clinical domains including mobility and emotional well-being [7]. Improving the transition from inpatient stroke rehabilitation to the community is identified as a high priority topic by the World Stroke Organization [8]. Early Supported Discharge (ESD) services were developed to improve the transition and facilitate community reintegration by accelerating inpatient discharge and providing an equivalent level of rehabilitation at home [9]. Although the content of an effective service is not clearly defined, it has been shown that ESD can reduce disability and long-term dependency [10]. However, a basic level of mobility such as the ability to stand up from a chair is critical for the possibility of an early discharge [11]. In addition, patients still experience an abrupt and disjointed care transition when professional support from an ESD team dwindles and caregivers would benefit from more support and training [12]. Hence, strategies to improve mobility in the first months poststroke and smoothen the care transition are needed to facilitate and complement current ESD services.

Training caregivers to become an exercise coach and perform Caregiver-Mediated Exercises (CME) may be a resource-efficient strategy to augment exercise therapy in the first 6 months poststroke [13]. Meta-analyses have shown that augmented exercise therapy and repetitive, progressive and task-oriented training can improve mobility and independence in Activities of Daily Living (ADL) in the first 6 months poststroke [14, 15]. In addition, involving caregivers in goal-setting and rehabilitation may smoothen the care transition by improving preparedness for discharge and enhancing self-efficacy in the home setting. A blended care approach, consisting of a combination of telerehabilitation and face-to-face support, is another promising paradigm to facilitate the transition from professional support to self-management [16]. Two recently conducted Randomized Controlled Trials (RCTs) showed that CME supported by an offline application with exercise videos was feasible, safe, and effective in improving psychological outcomes [17, 18]. In addition, dyads randomized to the CME intervention experienced a smooth transition to the home setting [19]. Unfortunately, the intervention did not improve self-reported mobility, nor were significant differences found for secondary outcomes of physical functioning. The neutral outcome may be explained by insufficient treatment contrast between the intervention and control group in terms of total exercise time, as a result of contamination. Interestingly, the Australian trial did find a reduction in Length Of Stay (LOS) in rehabilitation wards and a significant improvement of mobility and extended ADL in a per-protocol analysis of 20 patients that returned home during the intervention [17]. In addition to the CME program, this subgroup received telerehabilitation and home visits after discharge. Blended care and extending CME to the community, where caregivers can motivate the patient and provide continuity of exercises when professional support tapers off, could therefore be a promising addition to ESD services.

The Allied Rehabilitation using caregiver MEDiated exercises for Stroke (Armed4Stroke) study investigates a CME program using a blended care approach, in addition to usual care. Compared to previous trials, there is an increased focus on joint goal-setting and rehabilitation in the home situation to increase motivation and stimulate self-management. The training program is developed to achieve important milestones for community ambulation [20, 21] and dyads are supported by a web-based telerehabilitation system with individualized goals, exercise videos and a messaging environment to communicate with their physiotherapist.

The aim of this paper is to describe the Armed4Stroke study design following the SPIRIT Statement (Additional file 1: SPIRIT Checklist). The multicentre, observer-blinded trial aims to assess the effects of the Armed4Stroke program in addition to usual care, starting during the subacute phase poststroke. We hypothesize that the Armed4Stroke program will:

1. Increase patients’ self-reported mobility;
2. Smoothen the care transition, resulting in reduced LOS and better preparedness for discharge;
3. Improve psychological functioning of patients and caregivers, such as mood and self-efficacy.

## Methods

### Design

The Armed4Stroke study is designed as a multicentre, observer-blinded, phase II, randomized controlled superiority trial with two parallel groups. Patient-caregiver dyads will be randomly allocated at a 1:1 ratio to 8 weeks of CME and telerehabilitation (Armed4Stroke program) in addition to usual care, or usual care alone. The trial is registered since 11 December 2018 in the Netherlands Trial Register as NL7422. The study protocol was approved by the Medical Ethics Review Committee of VU Medical Centre on April 30th 2019 and is registered with trial number 2019.081 - NL67357.029.18.

### Setting

The multicentre study is conducted in the in- and outpatient clinics of 4 rehabilitation centres in the Netherlands: 1) Reade Rehabilitation, Amsterdam; 2) Roessingh, Centre for Rehabilitation, Enschede; 3) Sint Maartenskliniek, Nijmegen; and 4) Vogellanden, Zwolle. The Amsterdam UMC, location VU Medical Centre, is the initiator of this study in collaboration with Amsterdam Rehabilitation Research Centre | Reade and Roessingh Research and Development, Enschede.

### Participants

Seventy-two patients with stroke, and 1 or 2 of their informal caregivers will be enrolled during in- or outpatient rehabilitation. The caregiver can be a partner, family member, or other person close to the patient. They are not healthcare professionals, nor are they paid for their efforts. Stroke is defined by the World Health Organization as “a clinical syndrome typified by rapidly developing signs of focal or global disturbance of cerebral functions, lasting more than 24 hours or leading to death, with no apparent causes other than of vascular origin” [22]. Subarachnoid haemorrhage, and stroke resulting from a brain tumour or traumatic brain injury are excluded as these subtypes differ in their prognosis.

Inclusion criteria for both patient and caregiver are: 1) 18 years or older; 2) written informed consent; 3) able to understand the Dutch language on sufficient level to understand instructions and complete the questionnaires; and 4) motivated for CME. Inclusion criteria for the patient are: 1) < 3 months poststroke; 2) living independently prior to stroke; 3) discharged or planned to be discharged home; 4) able to follow instructions (Montreal Cognitive Assessment [MoCA] score > 21 points); and 5) able and willing to appoint an informal caregiver. An additional inclusion criterion for the caregiver is: being medically stable and able to support the patient. A trained physiotherapist will judge safety and determine if caregivers are physically and mentally able to support the patient during an intake exercise session. Exclusion criteria for both patient and caregiver will be a serious comorbidity that interferes with participation, e.g., premorbid restrictions in mobility as a result of a neurological disease, congestive heart failure or fractures of the lower extremity. Patients will not be enrolled in other clinical trials during the study period.

#### Baseline characteristics

Demographic and social characteristics of patients and caregivers will be recorded at baseline including: age, sex, country of birth, level of education, type and duration of relationship, living arrangement, work status, and comorbidities following the Cumulative Illness Rating Scale [23]. In addition, we will document stroke characteristics: type and location, stroke severity following the Bamford classification system [24], date of stroke, previous stroke, hemiplegic side, presence of sensory deficits, hemianopia, neglect or aphasia, communicative ability following item 19 of the Utrecht Communication Observation [25], and cognition following the MoCA [26].

### Study procedures

**Fig. 1:**
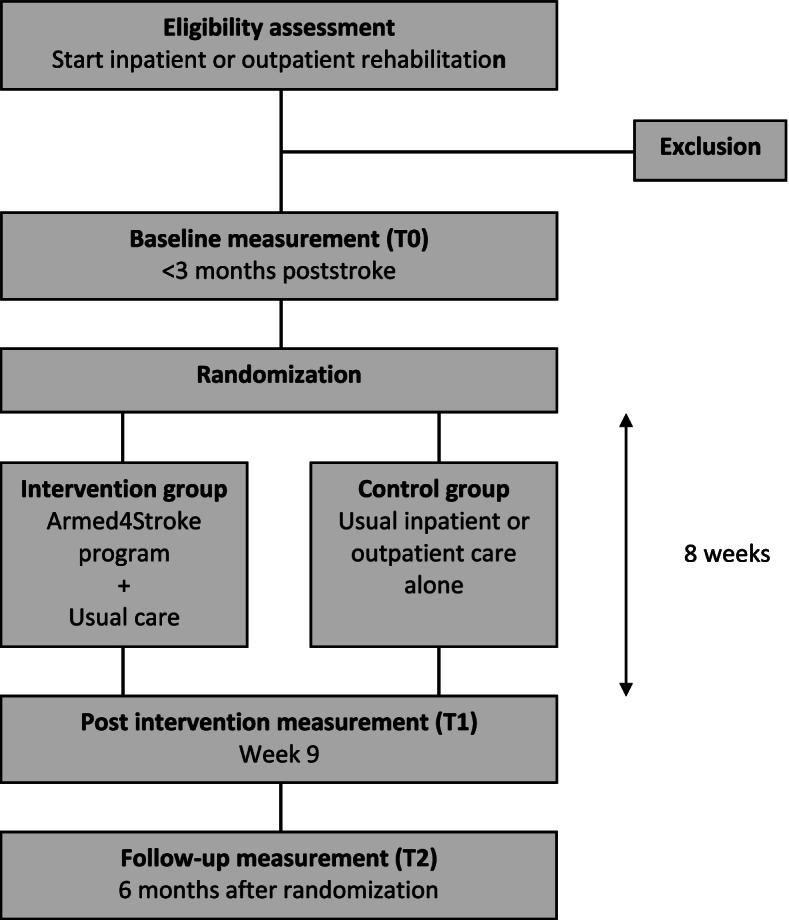
Study design

Every patient with stroke, starting in- or outpatient rehabilitation is screened for eligibility according to the inclusion and exclusion criteria by their rehabilitation physician and/or physiotherapist. Eligible dyads receive verbal and written information about the study. If a dyad wants to participate after a reflection period of 1 week, the intake exercise session is scheduled. Upon enrolment, informed consent will be signed by patient and caregiver(s) (Additional file 2: Informed consent form) and primary and secondary endpoints are measured by an independent assessor (MM, CN). Following the assessments, an activity monitor is worn by the patient for 1 week and dyads are randomized to the intervention or control group at a 1:1 ratio using an online randomization module with a minimization algorithm to prevent unequal group sizes. The module will be managed by a researcher (EW, GK) from the initiating institute, not involved in inclusion, assessment or intervention delivery. Factors in the minimization include: 1) centre; 2) inpatient versus outpatient; 3) ≥70 years versus < 70; and 4) Functional Ambulation Category (FAC) 5 versus FAC < 5. Primary and secondary endpoints are repeated by a blinded assessor (MM) at the end of treatment and 6 months follow-up (Fig. 1: Study design). During the study period, serious adverse events will be recorded and reported to the Medical Ethics Review Committee of VU Medical Centre. Data collection is done using electronic Case Report Forms (eCRFs) within the Good Clinical Practice-proof, cloud-based clinical data management platform Castor EDC [27]. The study is monitored by the independent clinical research bureau from VU medical centre. Univariate checks, e.g., range checks, and conditional variables are built-in the eCRFs. The first participant was enrolled on September 2nd 2019 and participants are currently being recruited and enrolled.

### Armed4Stroke intervention program

The Armed4Stroke program consists of 8 weeks of complementary exercises executed with a caregiver (CME), in addition to usual care. A trained physiotherapist compiles a tailor-made, progressive training program, containing personal goals and task-specific exercises focusing on balance, gait, outdoor activities and physical training. A total of 31 goals and 80 exercises according to evidence-based physical therapy guidelines [28] were developed to achieve important milestones for community ambulation, based on expert’s opinion and items of the hierarchical Mini-BESTest [29] and Rivermead Mobility Index (RMI) [30]. Dyads are asked to perform the exercises minimally 5 times a week for 30 min per session. This intensity is in line with current exercise guidelines [28] and was shown to be feasible in previous CME studies [17, 18]. Dyads are advised to execute their program outside usual training hours and during weekends, when patients are mostly inactive [31, 32].

Dyads are supported by a trained physiotherapist during at least 4 face-to-face sessions, scheduled every other week. During the first session, the program is explained in detail and explicit attention is given to information about the influence of physical activity and exercise on mood and health-related quality of life [33]. In addition, the role of caregivers and their benefits will be discussed. During subsequent sessions, the program is adapted according to the progress and personal goals of dyads, barriers are identified and addressed, and dyads are motivated to continue their exercise program. During the program, dyads are supported by exercise videos which are built into a web-based telerehabilitation system. Telerehabilitation poststroke should not be restricted to a time, place or device [16]. Therefore, the web-based system can be accessed on all devices with an internet connection including a computer, tablet or smartphone. In addition, dyads can communicate a-synchronically (i.e., exchange messages independent of time) with their physiotherapist. Therapists can use the telerehabilitation system to set-up goals and exercises, monitor compliance and progress, give feedback and motivate participants. Physiotherapists were extensively trained in applying the program to optimize standardization.

### Usual care

All participants will receive usual in- or outpatient physical therapy according to the Royal Dutch Guidelines of Physical Therapy [28]. Physical therapy sessions are designed to improve control of standing balance, physical condition, and walking competency. There are no restrictions with respect to content, time, or duration of therapy. Patients randomized to the control group will not have access to the Armed4Stroke telerehabilitation module and no explicit attention is given to the involvement of caregivers in goal-setting and rehabilitation. An 8-week self-reported exercise diary is kept by all the participants to monitor compliance, record fall incidents and telerehabilitation use in a usual care context.

### Outcome measures

#### Primary endpoint

The primary outcome is the mobility domain of the SIS version 3.0 [34], a stroke-specific questionnaire that evaluates self-reported health on 8 domains and a Visual Analogue Scale (VAS). Items are scored on a 5-point Likert scale and domain scores are calculated ranging from 0 to 100 [35]. The SIS has excellent clinimetric properties in English [34, 35] and Dutch [36]. The mobility domain consists of 9 questions about perceived ability to maintain balance, make transfers or walk in the community. The other 7 domains, the VAS and a composite physical domain are evaluated as secondary endpoints.

#### Secondary endpoints patient and caregiver

##### General Self-efficacy Scale (GSES)

The GSES is a valid questionnaire to evaluate general self-efficacy beliefs [37, 38]. The 10 items are rated on a 4-point scale and summed to produce a total score.

##### Preparedness for Caregiving Scale (PCS) and Transition Preparedness Scale (TPS)

The PCS is a reliable and valid tool to assess transition preparedness in caregivers, to assume their new role and to handle the stresses of caregiving [39]. The PCS was translated to Dutch using forward- and back-translations and a modified version was developed to assess transition preparedness in patients, the TPS (Additional file 3: Transition Preparedness Scale). Psychometric evaluation of both scales is currently being conducted by the authors.

##### Hospital Anxiety and Depression Scale (HADS) and Computer Adaptive Tests (CAT)

The HADS is a psychometrically robust measure to assess mood [40, 41]. The scale consists of two 7-item subscales for anxiety and depression. In addition, anxiety and depression items of the Dutch-Flemish PROMIS item bank are administered with CAT [42–44]. CAT dynamically selects the optimal number of questions and the best next question, based on responses to previous questions.

##### Fatigue Severity Scale (FSS)

The 9-item FSS is a reliable and valid instrument to assess fatigue. A mean item score is calculated ranging from 1 to 7 [45].

##### General functioning subscale of the McMaster Family Assessment Device (FAD-GF)

The FAD-GF is a reliable and valid questionnaire to determine general family functioning [46, 47]. A mean item score is calculated ranging from 1 to 4, based on 12 items. Perceptions of family functioning can differ and therefore the FAD-GF is administered to both patients and caregivers [48].

#### Secondary endpoints patient

##### Length of Stay (LOS)

LOS is defined as the number of days between admission and discharge from the rehabilitation centre and will only be considered as a secondary endpoint for patients enrolled during inpatient rehabilitation.

##### Stroke Self-efficacy Questionnaire (SSEQ)

The SSEQ is a reliable and valid measure of self-efficacy in relevant domains of functioning poststroke [49]. The SSEQ consists of 13 items with scores ranging from 0 to 10. Rasch analysis identified 2 dimensions: 1) Activity; and 2) Self-management [50]; and we will report these separate dimensions.

##### RMI

The RMI measures self-reported mobility poststroke with 14 questions and 1 observation [51]. A total score of 0 to 15 is calculated. Psychometric properties of the English and Dutch version [51–53] have been established.

##### FAC score

Functional walking ability is evaluated with the 0 to 5 FAC score, a reliable, valid and responsive scale poststroke [54]. An assessor rates the level of independence, regardless of the use of assistive devices.

##### Six-minute Walking Test (6MWT) and Five-meter Walk Test (5MWT)

The 6MWT [55, 56] and 5MWT [57, 58] are psychometrically robust tests to determine walking capacity poststroke. Distance walked during 6 min is recorded in meters. Mean gait speed is calculated in meters per second based on 3 repetitions. The 5MWT at a comfortable speed is recommended as the most responsive and convenient method of evaluating gait speed, when compared to fast speed or a 10-m distance [59].

##### Leg section of the Motricity Index (MI-leg)

The MI-leg is a reliable and valid tool to assess strength and range of motion of the paretic leg [60, 61]. The test consists of 3 joint movements, i.e., ankle dorsiflexion, knee extension, and hip flexion which are rated on a 0 to 33 scale and summed. When the maximum score of 99 is achieved, 1 point is added.

##### Berg Balance Scale (BBS)

The BBS measures balance during 14 tasks [62]. Items are rated on a 0 to 4 scale and a total score is calculated ranging from 0 to 56. Reliability, validity and responsiveness are established poststroke [62, 63].

##### MOX-2 activity monitor

Physical activity will be measured for 7 days during wake hours using the MOX-2. The MOX-2 is a small, lightweight device and will be worn on the non-paretic thigh using an adhesive plaster. It measures 3D-accelerations during daily activities and mean daily physical activity is calculated over 1 week.

##### Nottingham Extended Activities of Daily Living index (NEADL)

The NEADL is a 22-item reliable and valid test of extended ADL poststroke [30, 64, 65]. Rasch analysis did not support a unidimensional structure or polytomous scoring [65]. Therefore, the original dichotomous scoring method and domain scores: 1) Mobility; 2) Kitchen; 3) Household; and 4) Leisure will be used.

##### Community Ambulation Questionnaire (CAQ)

The CAQ measures the level of independent walking outside the home, i.e., without physical assistance or supervision [66]. Patients are categorized as: 1) Unable to walk outside unassisted; 2) Only ambulant around the house (e.g., as far as the letterbox); 3) Ambulant in the immediate environment (e.g., around the block); or 4) Independent community ambulation (e.g., to visit a friend or shop).

##### The EuroQol 5D (EQ-5D)

The 3-level version of the EQ-5D is a valid and reliable, generic measure of health-related quality of life poststroke [67, 68]. The instrument consists of 5 dimensions: 1) Mobility; 2) Self-care; 3) Usual activities; 4) Pain/Discomfort; and 5) Anxiety/Depression; and a VAS to assess perceived health.

##### Modified Rankin Scale (mRS)

The mRS is a widely used 0 to 5 global rating scale of disability poststroke. An assessor rates the level of independence in pre-stroke activities. The scale has good reliability and validity poststroke [69].

#### Secondary endpoints caregiver

##### Caregiver Strain Index (CSI) and Care-related Quality of Life instrument (CarerQoL)

Both the CSI [70–72] and the CarerQoL [73–75] are reliable and valid instruments to evaluate caregiver burden. The CSI consists of 13 items which are summed to produce a total score. The CarerQoL consists of seven 3-level burden dimensions and a 0 to 100 VAS measuring happiness.

### Power analysis

We expect a significant improvement of 10 points (10%) on the SIS mobility domain (mean 79.4, SD 14) in favour of the intervention group. We expect that minimally 30 patients are required per trial arm [76]. Including 20% drop-outs (for patients and caregivers), a sample size of 72 patient-caregiver dyads is needed to achieve sufficient statistical power of 80% using a two-tailed significance level alpha of *p* < 0.05. Currently, 26 participants have been enrolled in the ongoing trial.

### Data analyses

Baseline characteristics will be presented in mean and standard deviation or median and interquartile range, depending on the normality of data distributions judged by visual plot and Shapiro-Wilk tests. Between-group differences will be studied to determine whether groups are comparable at baseline using non-parametric Wilcoxon signed rank sum tests when the data is not normally distributed, or student t-tests for independent samples in the presence of normally distributed data. The study endpoints will be compared between the intervention and control group at different time points using linear mixed model analysis. Time since stroke, group allocation, minimization factors and baseline value of the outcome measure will be added to the longitudinal model. Intention-to-treat analysis will be applied and all hypotheses will be tested two-sided using a critical value of < 0.05.

## Discussion

The Armed4Stroke trial is a multicentre observer-blinded RCT that aims to investigate the effects of an improved CME program in addition to usual care on recovery of self-reported mobility poststroke. CME is a promising paradigm to improve psychological outcomes of patients with stroke and their caregivers. Unfortunately, two previous CME trials have failed to achieve sufficient treatment contrast in terms of augmented exercise time [17, 18]. As a result, these trials were unable to show an improvement on mobility and other outcomes of physical functioning poststroke. The current trial aims to continue CME after inpatient discharge, in a period when professional support tapers off, reducing the impact of contamination and increasing treatment contrast. We hypothesize that the improved Armed4Stroke program can increase self-reported mobility and independence, in addition to psychological improvements.

Although augmented exercise therapy can result in improved walking ability and independence in ADL [14, 15], it is critical to develop an active lifestyle to prevent deterioration and maintain physical function in the community [77]. Most people with stroke living in the community adopt a sedentary lifestyle [78] and even community-living people with only mild motor impairments, do not use their walking capacity to engage in physical activity [4]. The increased focus on personal goals and rehabilitation in a relevant context aims to heighten motivation. In addition, caregivers can provide patients with sustained support and motivate patients when professional support tapers off [5]. Hence, active involvement of caregivers in goal-setting and exercise therapy during the rehabilitation phase may promote long-term adherence to recommended physical activity levels and improve mobility outcome.

Telerehabilitation and CME may be resource-efficient strategies to augment exercise therapy and continue rehabilitation in the home setting. The current evidence suggests that telerehabilitation and CME are at least equally effective, when compared to usual care [13, 79–81]. However, we would like to stress the importance of using these strategies to augment or extend rehabilitation instead of replacing traditional rehabilitation. Rehabilitation should start with professional face-to-face therapy to establish a good professional relationship and to provide greater opportunity for progressive, goal-oriented treatment while ensuring safety. In addition, caregivers of patients with stroke need professional support during preparation for discharge and in the first few months of adjustment at home [82]. A blended care approach which combines telerehabilitation with face-to-face support may empower and better prepare patient-caregiver dyads for inpatient discharge and facilitate the transition from face-to-face therapy to self-management at home.

The Armed4Stroke trials investigates a complex rehabilitation intervention with multiple interacting components and types of support. Physiotherapists are extensively trained in applying the Armed4Stroke program and preparing patient-caregiver dyads for their roles in the intervention to optimize standardization, promote adherence to the multifaceted content and ensure an optimal quality of intervention delivery. However, healthcare policies and resources in terms of financial support and staffing can challenge implementation of these complex rehabilitation trials. Professional support, either face-to-face or using telerehabilitation, after discharge may not be reimbursed by health insurance. In addition, insufficient staffing and resources of rehabilitation centres can be barriers for both recruitment and intervention delivery. Another barrier for recruitment could be the absence of an informal caregiver to participate in CME. Although our definition of an informal caregiver is broad, the current intervention may not be suitable for patients with a limited social support system.

Several limitations of our design should be addressed. First, the aim of the current trial does not include an evaluation of cost-effectiveness. Theoretically, telerehabilitation and CME can be used to augment exercise therapy and provide sustained support after discharge with limited resource-use. However, there is currently insufficient evidence about the cost-effectiveness of these strategies [13, 79]. Although no formal economic evaluation is planned, the protocol did include several secondary endpoints which can be used to evaluate cost-effectiveness including LOS, the EQ-5D and CarerQoL. Second, patient-caregiver dyads and physiotherapists cannot be blinded to treatment allocation. Therefore, our self-reported outcome measures may be subject to bias. However, the objective endpoints will be measured by a blinded assessor. Third, lack of blinding can result in contamination in terms of altered behaviour of the control group (i.e., CME and independent exercise time) and professionals (i.e., professional exercise time and telerehabilitation use). Therefore, the level of contamination will be monitored during the trial. Finally, we excluded patients with severe cognitive and/or communicative abilities to ensure that instructions are understood, safety is secured, and self-reported questionnaires can be completed. Therefore, treatment effects may not be generalizable to more severely affected patients.

## Conclusion

In conclusion, the Armed4Stroke clinical trial investigates the effects of an improved CME program with an increased focus on personal goals, rehabilitation in the home situation, and combined with web-based telerehabilitation services poststroke. The Armed4Stroke study is an important next step before proceeding to a larger phase III/IV cost-effectiveness trial.

## Supplementary Information

**Additional file 1.** SPIRIT checklist.

**Additional file 2.** Informed consent form.

**Additional file 3.** Transition Preparedness Scale.

## Data Availability

The datasets generated and/or analysed during the current study are available from the corresponding author on reasonable request. Final trial results will be communicated to the funding body in a brief report, to participants in a newsletter, to relevant healthcare professionals and other relevant groups during a congress or symposium and via a future scientific publication.

## Abbreviations

5MWT: 5 Meter Walk Test
6MWT: 6 Minute Walking Test
ADL: Activities of Daily Living
BBS: Berg Balance Scale
CAQ: Community Ambulation Questionnaire
CarerQoL: Care-related QoL
CAT: Computer Adaptive Tests
CME: Caregiver-Mediated Exercises
CSI: Caregiver Strain Index
EQ-5D: EuroQoL 5D
ESD: Early Supported Discharge
FAC: Functional Ambulation Category
FAD-GF: McMaster Family Assessment Device - General Functioning subscale
FSS: Fatigue Severity Scale
GSES: General Self-Efficacy Scale
HADS: Hospital Anxiety and Depression Scale
LOS: Length Of Stay
MI-leg: Leg section of the Motricity Index
MoCA: Montreal Cognitive Assessment
mRS: Modified Rankin Scale
NEADL: Nottingham Extended Activities of Daily Living index
PCS: Preparedness for Caregiving Scale (PCS)
RCT: Randomized Controlled Trial
RMI: Rivermead Mobility Index
SIS: Stroke Impact Scale
SSEQ: Stroke Self-Efficacy Questionnaire
TPS: Transition Preparedness Scale
VAS: Visual Analogue Scale
