# Supplementary material for: Can telerehabilitation services combined with caregiver-mediated exercises improve early supported discharge services poststroke? A study protocol for a multicentre, observer-blinded, randomized controlled trial
Source: BMC Neurol. 2022 Jan 17;22:29. doi: 10.1186/s12883-021-02533-w (PMC8762867; doi:10.1186/s12883-021-02533-w)
Supplement: Supplementary file 2 — Additional file 2. Informed consent form. [file 12883_2021_2533_MOESM2_ESM.docx]

**Informed consent form**

*ARMED4STROKE: Exercising with an informal caregiver during rehabilitation after stroke.*

- I have read the participant information. I could ask questions. My questions have been sufficiently answered. I had enough time to decide whether to participate.
- I know that participation is voluntary. I also know that I can decide not to participate or withdraw from participation at any time. I don’t have to give a reason for withdrawal.
- I know that my data must be stored for 15 years in Reade and Amsterdam UMC.
- I give permission to inform my rehabilitation physician about my participation in this study.
- I give permission for the collection and use of my data to answer the research question of this study.
- I consent to the exchange of data with researchers from other participating research locations.
- I know that some people may get access to my data for the purpose of auditing this study. These people are listed in the participant information. I give permission for access to these persons.
- I give permission to store my personal data for longer and to use it for future research in the field of stroke rehabilitation:

□ **yes**

□ **no**

- I give permission to contact me again for follow-up research after the end of this study:

□ **yes**

□ **no**

- I give permission to collaborate and exchange data with researchers from external institutions, and with researchers outside the European Union:

□ **yes**

□ **no**

- I want to participate in this study.

Name participant:

Signature: Date: __ / __ / __

- I declare that I have fully informed the participant about this study.
- If new information that could influence the participant’s consent, becomes available during the study, I will inform him or her in good time.

Name assessor (or its representative):

Signature: Date: __ / __ / __

*The participant receives a participant information letter and a copy of the signed informed consent form.*
