# Supplementary material for: Can telerehabilitation services combined with caregiver-mediated exercises improve early supported discharge services poststroke? A study protocol for a multicentre, observer-blinded, randomized controlled trial
Source: BMC Neurol. 2022 Jan 17;22:29. doi: 10.1186/s12883-021-02533-w (PMC8762867; doi:10.1186/s12883-021-02533-w)
Supplement: Supplementary file 3 — Additional file 3. Transition Preparedness Scale. [file 12883_2021_2533_MOESM3_ESM.docx]

**The Transition Preparedness Scale**

We know that people may feel well prepared for some aspects of functioning at home, and not as well prepared for other aspects. We would like to know how well prepared you think you are to do each of the following activities, even if you are not doing that type of activity now.

| How well prepared do you think you are … | **Not at all prepared** | **Not too well prepared** | **Somewhat well prepared** | **Pretty well prepared** | **Very well prepared** |
| --- | --- | --- | --- | --- | --- |
| 1. To take care of your physical needs? | 0 | 1 | 2 | 3 | 4 |
| 2. To take care of your emotional needs? | 0 | 1 | 2 | 3 | 4 |
| 3. To find out about and set up services for yourself? | 0 | 1 | 2 | 3 | 4 |
| 4. For discharge and functioning at home? | 0 | 1 | 2 | 3 | 4 |
| 5. To make daily activities feasible for both you and your family member? | 0 | 1 | 2 | 3 | 4 |
| 6. To respond to and handle emergencies? | 0 | 1 | 2 | 3 | 4 |
| 7. To get the help and information you need from the healthcare system? | 0 | 1 | 2 | 3 | 4 |
| 8. Overall, to take care of yourself? | 0 | 1 | 2 | 3 | 4 |
| 9. Is there anything specific you would like to be better prepared for? | | | | | |
